# Genome Sequences of 17 Pasteurella multocida Strains Involved in Cases of Rabbit Pasteurellosis

**DOI:** 10.1128/MRA.00681-19

**Published:** 2019-09-12

**Authors:** Florent Kempf, Emilie Chambellon, Emmanuelle Helloin, Hervé Garreau, Frédéric Lantier

**Affiliations:** a ISP, INRA, Université François Rabelais de Tours, Nouzilly, France; b GenePhySE, INRA, Castanet Tolosan, France; University of Arizona

## Abstract

This article reports draft genome sequences of 17 Pasteurella multocida strains isolated from naturally infected rabbits. The total lengths of the assembled contigs ranged between 2.21 and 2.48 Mb, and the total number of genes detected on the contigs ranged between 2,088 and 2,416.

## ANNOUNCEMENT

Pasteurella multocida can be isolated as a pathogen or a commensal organism from a wide spectrum of animal hosts (multocida literally means “multikiller”). Several forms of severe pasteurellosis have been described in domestic animals and may cause important economic losses (1). In particular, rabbit pasteurellosis is a disease of concern in countries where rabbits are bred on a large scale for human consumption (2). Comparative genomics may help in understanding the genetic relationships between host-associated populations.

Here, we have considered 17 strains of P. multocida isolated from independent cases of pasteurellosis affecting rabbit breeding units in main production regions in France (Table 1). Their identity was confirmed by the INRA Centre for Microbial Resources-Bacterial Pathogens (CIRM-BP) through appropriate biochemical tests (3) and a specific pair of PCR primers (KMT1T7 and KMT1SP6) (4). They were stored in liquid nitrogen. Prior to genome sequencing, strains were incubated for 8 hours in 2 ml of brain heart infusion (BHI) medium before being plated onto Trypticase soy agar medium supplemented with yeast extract (TSA-YE; Difco, Bordeaux, France) and 5% horse serum (GIBCO-BRL, Grand Island, NY, USA). After strains were incubated overnight, 10 colonies were picked and plated onto a slope with TSA-YE and 5% serum. All cultures were performed at 37°C under 5% CO_2_ conditions.

**TABLE 1:** Characteristics and genome accession numbers of 17 Pasteurella multocida strains

Strain	Type of read	No. of reads	Coverage (×)	*N*_50_ value (bp)	No. of contigs	Total sequence length (bp)	G+C content (%)	No. of genes	SRA accession no.	GenBank accession no.
CIRMBP-0747	Illumina	8,243,412	710.0	210,043	16	2,137,782	40.2	2,168	SRS1876742	MTIE00000000
CIRMBP-0749	Illumina	7,174,231	760.0	562,113	15	2,335,189	40.3	2,250	SRS1876749	MTEA00000000
CIRMBP-0758	Illumina	9,034,841	655.0	197,921	30	2,407,798	40.2	2,344	SRS1876752	MTEB00000000
CIRMBP-0760	Illumina	7,799,183	830.0	190,835	31	2,400,855	40.2	2,335	SRS1876753	MTEC00000000
CIRMBP-0782	Illumina	7,846,088	265.0	352,828	12	2,270,275	40.2	2,166	SRS1876746	MTED00000000
CIRMBP-0783	Illumina	6,079,082	550.0	313,092	16	2,332,744	40.3	2,249	SRS1876745	MTEF00000000
CIRMBP-0812	Illumina	8,061,249	705.0	333,581	16	2,335,382	40.3	2,251	SRS1876757	MTDZ00000000
CIRMBP-0817	Illumina	7,951,986	700.0	216,212	38	2,431,039	40.2	2,371	SRS1876754	MTEE00000000
CIRMBP-0827	Illumina	7,714,421	690.0	215,414	36	2,374,729	40.2	2,311	SRS1876750	MTIG00000000
CIRMBP-0835	Illumina	4,596,700	390.0	199,937	21	2,271,558	40.2	2,172	SRS1876743	MTIF00000000
CIRMBP-0872	Illumina	7,600,667	700.0	276,902	15	2,214,585	40.3	2,088	SRS1876758	MTIH00000000
CIRMBP-0873	PacBio	46,846	196.0	16,177	3	2,480,901	40.4	2,410	SRS1876751	CP020347, CP020348, CP020349
CIRMBP-0877	Illumina	8,358,588	340.0	190,235	29	2,349,029	40.2	2,266	SRS1876756	MTII00000000
CIRMBP-0884	PacBio	16,348	80.0	18,934	2	2,463,037	40.5	2,416	SRS1876744	CP020345, CP020346
CIRMBP-0906	Illumina	8,903,970	815.0	276,842	15	2,270,271	40.2	2169	SRS1876755	MTIJ00000000
CIRMBP-0922	Illumina	7,119,199	640.0	210,110	14	2,275,582	40.2	2,173	SRS1876748	MTIK00000000
CIRMBP-0927	Illumina	4,943,632	400	353,268	20	2,278,436	40.2	2,180	SRS1876747	MTIL00000000

Each slope was rinsed with 3 ml of physiological saline to make bacterial suspensions; 1 ml of each suspension was kept to perform genomic DNA extractions using silica-based columns (NucleoBond AXG; Macherey-Nagel, Hoerdt, France).

Two strains, namely, CIRMBP-0873 and CIRMBP-0884, were sequenced using single-molecule real-time technology (PacBio RS II; GATC Biotech, Inc., Constance, Germany). *De novo* assembly of PacBio reads was performed following the Hierarchical Genome Assembly Process 3 (HGAP3) procedure (5), including an initial filtering step of polymerase reads and subreads, followed by a draft assembly performed through Celera (6), and final assembly polishing with Quiver (5). We did not detect any short inserts. Three and two contigs were obtained for CIRMBP-0873 and CIRMBP-0884, respectively (Table 1).

Fifteen additional strains were sequenced through the Illumina HiSeq 2500 platform using a paired-end sequencing approach. The average genome coverage exceeded 200× for all strains (Table 1). Read quality control, adapter removal, deduplication, and final filtering were performed using FastQC (7), Trimmomatic (8), FastUniq (9), and IlluminaUtils (10), respectively. *De novo* assemblies were performed with SPAdes 3.10.1 (11) using the following parameters: –sc; -k 21, 33, 55; and –careful. The resulting draft genomes included between 12 and 38 contigs (size, >1 kb) and presented *N*_50_ sizes ranging from 190,235 bp to 586,840 bp (Table 1).

The 17 draft genomes resulting from PacBio or Illumina sequencing were annotated using the Prokaryotic Genome Annotation Pipeline (PGAP) (12). The number of potential genes ranged from 2,088 to 2,416 (Table 1).

### Data availability.

The genome sequences have been deposited in NCBI GenBank under BioProject accession number PRJNA355236 and the accession numbers presented in Table 1.
